# Spatiotemporal Temperature Distribution of NIR Irradiated Polypyrrole Nanoparticles and Effects of pH

**DOI:** 10.3390/polym14153151

**Published:** 2022-08-02

**Authors:** Omar Peñuñuri-Miranda, Miguel Olivas-Martinez, José Alberto Ibarra-Espinoza, Rosalva Josefina Rodríguez-Córdova, Karol Yesenia Hernández-Giottonini, Daniel Fernández-Quiroz, Paul Zavala-Rivera, Armando Lucero-Acuña

**Affiliations:** 1 Department of Chemical and Metallurgical Engineering, University of Sonora, Hermosillo 83000, Mexico; a212205033@unison.mx (O.P.-M.); miguel.olivas@unison.mx (M.O.-M.); a217201103@unison.mx (J.A.I.-E.); rosalva.rodriguez@unison.mx (R.J.R.-C.); yesenia.hgio@hotmail.com (K.Y.H.-G.); daniel.fernandez@unison.mx (D.F.-Q.); paul.zavala@unison.mx (P.Z.-R.); 2 Nanotechnology Graduate Program, Department of Physics, University of Sonora, Hermosillo 83000, Mexico

**Keywords:** polypyrrole nanoparticles, NIR laser irradiation, photothermal modeling, photothermal transduction efficiency, overall heat transfer coefficient

## Abstract

The spatiotemporal temperature distributions of NIR irradiated polypyrrole nanoparticles (PPN) were evaluated by varying PPN concentrations and the pH of suspensions. The PPN were synthesized by oxidative chemical polymerization, resulting in a hydrodynamic diameter of 98 ± 2 nm, which is maintained in the pH range of 4.2–10; while the zeta potential is significantly affected, decreasing from 20 ± 2 mV to −5 ± 1 mV at the same pH range. The temperature profiles of PPN suspensions were obtained using a NIR laser beam (1.5 W centered at 808 nm). These results were analyzed with a three-dimensional predictive unsteady-state heat transfer model that considers heat conduction, photothermal heating from laser irradiation, and heat generation due to the water absorption. The temperature profiles of PPN under laser irradiation are concentration-dependent, while the pH increase only induces a slight reduction in the temperature profiles. The model predicts a value of photothermal transduction efficiency (η) of 0.68 for the PPN. Furthermore, a linear dependency was found for the overall heat transfer coefficient (U) and η with the suspension temperature and pH, respectively. Finally, the model developed in this work could help identify the exposure time and concentration doses for different tissues and cells (pH-dependent) in photothermal applications.

## 1. Introduction

Nanoparticles based on conductive polymers have emerged as a promising material for applications in targeted photothermal medical therapies [1,2]. In this therapy, nanoparticles could be placed at different tissue-penetration depths and convert irradiated energy from the near-infrared (NIR) region into thermal energy [3,4]. Several conductive polymers, such as polyaniline, polypyrrole, etc., could be used for photothermal therapies due to their biocompatibility and excellent photothermal conversion performance [5,6,7,8]. Within these conductive polymers, polypyrrole is one of the most widely used for developing nanomaterials for biomedical applications [9]. Furthermore, polypyrrole-based nanostructures are being studied because of their strong absorption in the NIR region (λ = 700–1200 nm) [2]. For instance, polypyrrole nanoparticles (PPN) could generate heat suitable to ablate cancer cells through their effective photon-to-thermal energy transfer [10,11,12]. Moreover, PPN could be used singly or combined with chemotherapy agents [11].

Furthermore, PPN shows high specificity and excellent anti-cancer effects in cellular and animal experiments [13]. The elevated temperature could thus target cancer cells while avoiding significant side effects on normal cells. The latter is based on the higher heat tolerance of normal cells compared to cancer cells [14]. However, the photothermal application of polypyrrole-based materials is still in its early stages. Further research is required to understand and improve its potential performance under diverse in vitro or in vivo scenarios. In this context, one of the most common PPN preparation methods is oxidative chemical polymerization, consisting of a facile one-step aqueous dispersion polymerization that could be used with poly(vinyl alcohol) as stabilizing agent [15]. This method allows, up to some degree, the tuning of PPN properties by changing the stabilizing agent, varying the stabilizing agent concentration, and adjusting the molar ratios of the oxidative agent and the pyrrole monomer [16].

The modeling of heat dissipation from suspended nanoparticles irradiated at different wavelengths has been reported previously [17,18,19]. Roper et al. determined the efficiency of transducing resonant continuous-wave irradiation to bulk heat by a gold NP suspension by analyzing thermal energy transfer in a thermally isolated system with a macroscopic linearized heat transfer model that incorporates radiation and conduction [20]. The calculation of the photothermal transduction efficiency requires theoretical analysis of the experimental data [20,21,22,23]. Huang et al. analyzed the temperature distribution with a two-dimensional model for either fluid or tissue containing gold nanorods, based on the Pennes’ bioheat equation, with an additional term to account for energy released by the gold nanorods [24].

In this work, the chemical oxidative polymerization technique was used to prepare PPN, and their properties at different pH values were studied. Furthermore, the spatiotemporal temperature distributions of NIR-irradiated PPN were experimentally evaluated under different particle concentrations and pH values. An unsteady-state three-dimensional heat transfer model that describes the temperature behavior of the suspensions by considering conduction, photothermal heating from laser irradiation, and heat generation due to the water absorption was developed. The model was validated with experimentally obtained temperature measurements of PPN suspensions irradiated in the NIR region. This mathematical approach allows the prediction of the nanoparticle spatiotemporal temperature distributions and could help improve the doses of bioactive NPs and times of light irradiation at different pH values to reach the desired temperatures in photothermal therapies.

## 2. Materials and Methods

### 2.1. Materials

Pyrrole (reagent grade, 98%), iron (III) chloride hexahydrate (reagent grade, 97%, FeCl_3_·6H_2_O), and polyvinyl alcohol (86.7–88.7% hydrolysis, Mw~31,000 a.m.u, PVA) were purchased from Sigma Aldrich, Inc., St. Louis, MO, USA. The pyrrole monomer was purified by distillation under vacuum and inert atmosphere conditions before use.

### 2.2. Synthesis of PPN

**Figure 1:**
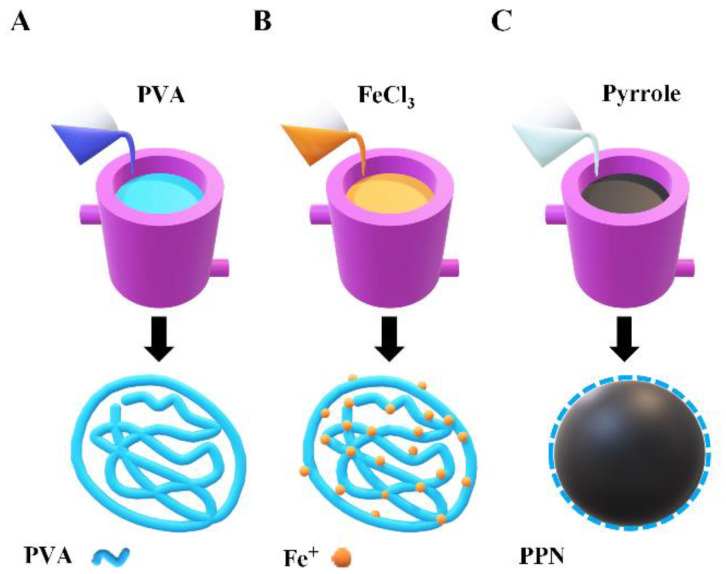
PPN synthesis scheme by chemical oxidation of pyrrole. (**A**) PVA solution at 5 °C; (**B**) formation of PVA/iron complex, and (**C**) polymerization of pyrrole and formation of PPN.

PPN were synthesized by the oxidative chemical polymerization of pyrrole using ferric chloride as oxidant and PVA as stabilizer [15,25,26]. A schematic of the synthesis of PPN is presented in Figure 1. A total of 20 mL of an aqueous solution of 7.5% of PVA (% w/v) was kept under magnetic stirring at 500 pm, at 5 °C (Figure 1A). Then, 4.6 mmol of ferric chloride ([FeCl_3_]/[monomer] = 2.3) was added to a PVA solution. The mixture reacts for 1 h to reach equilibrium, forming the PVA/iron cation complex (orange solution) (Figure 1B). Then, 2 mmol of pyrrole monomer was added to the PVA/iron cation complex solution. The solution turned black-green, indicating that polymerization started (Figure 1C), and the polymerization process was allowed to react for 4 h with continuous stirring and at 5 °C. Then, PPN was washed by three centrifugation cycles at 22,096× *g* (14,500 rpm) for 35 min, where supernatant was discarded, and the precipitate was resuspended in deionized water. Finally, PPN was collected and stored at 4 °C for further use. All experiments were performed in triplicate.

### 2.3. pH Effects over PPN

The polymerization of pyrrole with FeCl_3_ and PVA results in acidic PPN suspensions. Accordingly, the pH values of the PPN suspensions must be tuned to be used in biological applications. Therefore, it is necessary to evaluate the influence of pH on the properties of the PPN. The pH values studied were 4.2 (pH obtained from synthesis), 6, 7.4, 8, and 10; adjusted using solutions of 0.1 M NaOH.

### 2.4. Characterization of PPN

Hydrodynamic size, polydispersity index (PDI), and zeta potential (ζ) were measured using a Zetasizer (Zetasizer Nano ZS Malvern Instruments Ltd., Worcestershire, UK). Hydrodynamic size measurements of PPN were performed by the Dynamic Light Scattering technique (DLS). A refractive index of 1.33 and water as a dispersant were used in the analysis. Each sample was measured three times with at least 10 runs at 25 °C for the size and ζ analysis. The ζ was obtained by using the laser Doppler electrophoresis technique. The morphology of PPN was observed with a field transmission electron microscopy (TEM) JEOLTEM-2010F with an operating voltage of 200 kV (JEOL, Ltd., Tokyo, Japan). For TEM observations, a drop of the sample solution was spread on a smooth film of a carbon-coated copper TEM grid and dried. After that, 1% phosphotungstic acid was applied on the grid for 1 min; the excess was absorbed using filter paper. All samples were subsequently dried in a vacuum before observation. The optical absorption spectra of PPN were acquired in a UV6300PC spectrophotometer (VWR, Radnor, PA, USA). The characteristic absorption band of PPN in the NIR region was verified. A standard curve of PPN suspensions was built at 808 nm to determine the PPN concentration. All experiments were performed in triplicate.

### 2.5. Photothermal Evaluation of PPN

**Figure 2:**
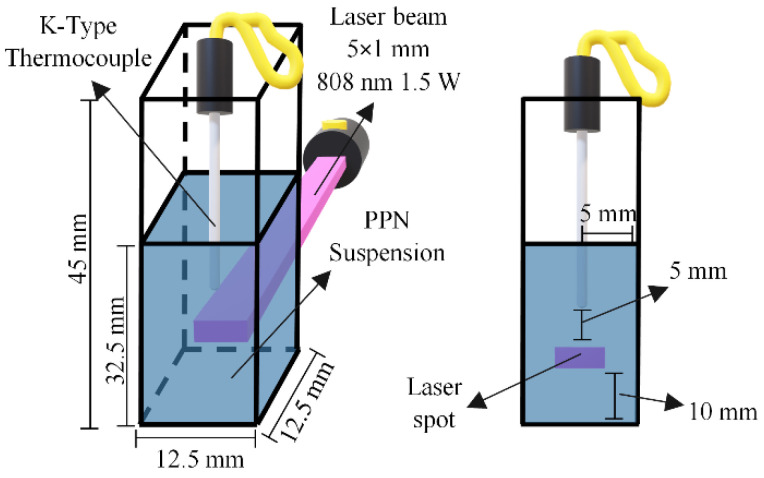
Scheme of heating experiments with the laser irradiation, indicating the dimensions of the quartz cuvette cell and the suspension volume (**left**), and a frontal view with the approximate position of the laser spot and the thermocouple (**right**).

The temperature variations of PPN suspensions irradiated with an NIR laser beam with an output power of 1.5 W centered at 808 nm (Changchun New Industries Optoelectronics Tech Co., Ltd., Model No. PSU-III-LED, Changchun, China) were measured. PPN suspensions were placed into a rectangular quartz cuvette cell and irradiated with the NIR laser. The experimental setup for the analysis and data acquisition is shown in Figure 2. The temperature variations were measured using a K-Type thermocouple connected to a multimeter that acted as a data acquisitor. Suspensions of PPN from 0 to 30 µg/mL and the pH of nanoparticle suspensions from 4.2 to 10 were analyzed. As a control, the temperature of a nonirradiated cuvette cell with water was measured simultaneously to calculate the temperature increments of the suspensions; that is, to adjust to the ambient temperature variations.

### 2.6. Spatiotemporal Temperature Distribution Analysis

The spatiotemporal temperature distribution of irradiated PNP suspensions was mathematically analyzed by modeling PPN suspensions inside a quartz cuvette. The modeled PNP suspension volume is comprised of two regions: the laser region, where the laser irradiates and the heat is produced, then the suspension region, where the heat is transported only by heat conduction. The energy equation in the laser region is:

(1) $$\rho C_{P}\frac{\partial T}{\partial t}\;=\;k\nabla^{2}T\;+\;\eta \frac{I}{V}\left(1-10^{-OD}\right)\;+\;S_{W}\;$$

where the term on the left side is the thermal energy accumulation. The first term on the right-hand side is the heat conduction, and the second term represents the photothermal heating from laser irradiation. This second term $\eta \frac{I}{V}\left(1-10^{-OD}\right)$ consists of heat generation due to the PPN. Furthermore, the heat generation due to the water absorption is considered in the third term of the right side ($S_{w}$). The energy equation for the suspension region is

(2) $$\rho C_{P}\frac{\partial T}{\partial t}=k\nabla^{2}T\;$$

The equation shows the accumulation term on the left-hand side, and on the right-hand side, only the conduction term appears.

In these equations ρ (kg/m^3^) is the density and $C_{P\;}$ (J/kg·K) is the heat capacity of the suspension, respectively, k is the thermal conductivity (W/m·K), η counts for the photothermal transduction efficiency, I (W) is the laser power, V (m^3^) stands for the irradiated volume, OD is the optical density of the suspension and $S_{W}$ (W/m^3^) is the water heat generation in the irradiated volume.

To solve Equations (1) and (2), the following boundary condition was applied to the cell walls and the air/suspension interface:

(3) $$q\;=\;U\left(T_{f}-T\right)+\varepsilon \sigma \left(T_{\infty }^{4}-T^{4}\right)$$

where q is the heat flux at the system boundaries (W/m^2^), U is the overall heat transfer coefficient (W/m^2^·K), $T_{f}$ is the temperature of media surrounding the cell (air) (K), T is the suspension temperature (K), ε is the emissivity of either the cell wall or the air/solution interface, σ is the Stefan–Boltzmann constant, and $T_{\infty }$ is the temperature of the radiation sink on the exterior of the domain (K). The overall heat transfer coefficient U incorporates the thermal resistance of the cell wall and the external free convection thermal resistance. Furthermore, the bottom of the quartz cuvette was considered adiabatic, resulting in the following boundary condition:

(4) q = 0 at z = 0

The main physical parameters used in the mathematical model were obtained from the experimental setup and the literature [27], as presented in Table 1. The model was numerically solved using Ansys Fluent R3 [20,21] using a hexahedral mesh of 85,536 elements. A semi-implicit method for pressure-linked equations was used with a time step of 0.4 s and 3750-time elements.

#### Determination of Model Parameters: η, U, and $S_{W}$

The values of parameters η, U and $S_{W}$ were obtained by fitting the model temperature predictions to the experimental measurements during laser heating. A two-step approach was followed: first, the order of magnitude η and U was obtained by analyzing a hypothetical case of the adiabatic cell and using a heat transfer correlation, respectively. The second step consisted of minimizing the sum of squares of differences between the experimental and predicted temperature increments using the heat model.

The order of magnitude of η was determined from the maximum temperature in the irradiated system (analyzed by considering a hypothetical case of an adiabatic cell), where all the energy absorbed by the system is accumulated, resulting in the maximum possible temperature at the given laser power and time of irradiation. The value of η defined as baseline must satisfy two conditions at a specific PPN concentration. The first condition is that the temperature increment in the adiabatic case be higher than the experimental one. The second condition is that the initial heating rate be faster than the experimental heating rate (Figure S1).

The order of magnitude of U was obtained using a correlation for free convection on a vertical surface. The properties of air used were estimated from an average temperature between the temperature of the surrounding media and the temperature measured by the thermocouple in the heating plateau (Figure S2).

The second step for determining η and U was implemented as follows: a set of values of U for each concentration and a single value of η equal to or greater than the baseline values were used to solve the model. Then the model was solved using the parameters and conditions described above. Next, the sum of squares was calculated for the values following Equation (5). The process was repeated at least three times to find the values of η and U and the minimum sum of squares. The term $S_{W}$ was obtained by fitting the heat model to the experimental data at a concentration of 0 µg/mL nanoparticles.

(5) $$\sigma^{2}=\overset{n}{\underset{i=1}{\sum }}\frac{{\left(\Delta T_{exp}-\Delta T_{sim}\right)}^{2}}{n-k}$$

### 2.7. Statistical Data Analysis

The statistical analysis for PPN hydrodynamic size, PDI, and ζ measurements was performed by the software OriginPro (ver. 9). Results were analyzed by ANOVA followed by Tukey’s HSD test (α = 0.05). The differences were considered statistically significant when the *p*-values were minor or equal to 0.05.

## 3. Results and Discussion

### 3.1. Characterization of PPN Synthesis

**Figure 3:**
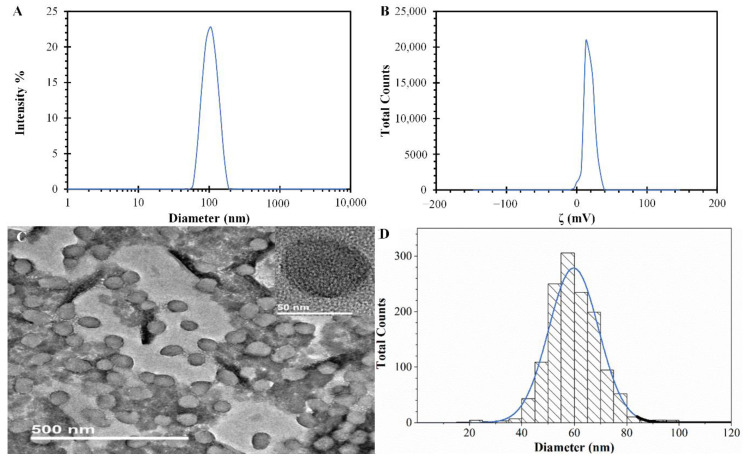
PPN properties were obtained from the synthesis (pH value from the synthesis of 4.2). (**A**) Diameter size distribution from DLS measurements. Means ± SD, *n* = 3. (**B**) ζ distribution. Means ± SD, *n* = 3. (**C**) TEM image of PPN with an insert of a single particle. (**D**) Histogram of the size distribution of PPN from TEM images (analysis with a number of particles >700).

PPN was synthesized by chemical oxidizing polymerization with hydrodynamic average sizes of 98 ± 2 nm from three independent preparations, verifying the method’s reproducibility. Furthermore, the DLS results show a narrow size distribution with a PDI of 0.04 ± 0.02 (as shown in Figure 3A). Different sizes can be obtained by varying the concentrations and molecular weight of PVA and the molar ratio between FeCl_3_ and pyrrole monomer [16]. Furthermore, using a different stabilizer in the chemical oxidizing polymerization could influence the PPN sizes. The literature has reported polyvinylpyrrolidone (PVP) as a stabilizer, resulting in PPN sizes ranging from 151.5 to 93.5 nm in the function of the PVP concentration increment [28]. The ζ distribution of PPN that resulted from the synthesis shows a single peak with a narrow width and a positive value, as seen in Figure 3B. This value, obtained from three independent preparations of PPN, was 20.0 ± 2.1 mV, indicating good nanoparticle stability due to electrostatic particle repulsion and preventing further particle aggregation [29]. The positive charge of these PPN is attributed to the presence of amino groups in the polypyrrole polymeric chain [30]. Other authors using a similar synthesis method of PPN report ζ values of 14.0 ± 0.4 mV for particles of 106 ± 1 nm [31]. The slight differences in the ζ values may be due to the concentrations of PVA used in the synthesis since the PPN surface covered by this stabilizer changes with the concentration used in the synthesis. Then, as the PVA concentration increases in the synthesis, the PPN surface-exposed hydroxyl groups increase, producing variations in the PPN surface charge.

The PPN morphologies and sizes can be observed in Figure 3C,D, respectively. The PPN has a uniform quasi-spherical morphology, with diameters of ~60 nm, as shown in Figure 3C and the insert. These morphologies and sizes agree with previous literature reports [32]. Furthermore, the excellent monodispersity can be confirmed by the narrow histogram distribution presented in Figure 3D, obtained from TEM pictures using the free software ImageJ version 1.53e. The difference in the sizes obtained from the DLS technique and the TEM analysis, as known, is related to the hydrodynamic size of PPN in the suspension (DLS) and the actual size of dried particles (TEM). Furthermore, in the literature, it was found that PPN synthesized with different methods showed similar amorphous structures [33,34].

**Figure 4:**
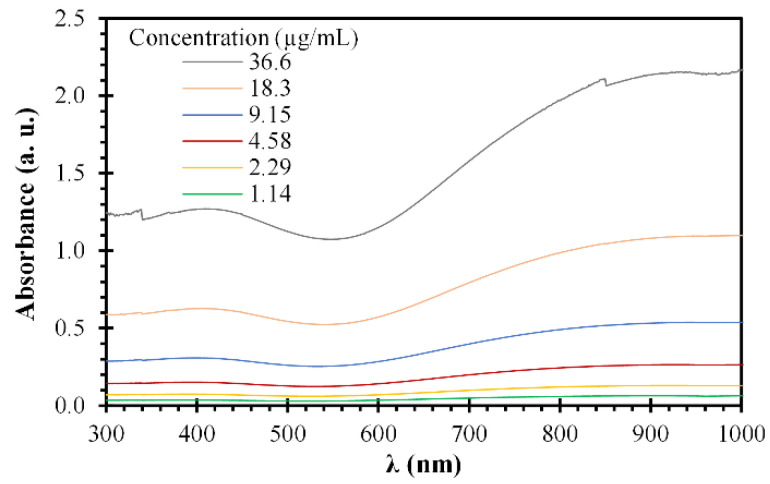
Optical absorption spectra of PPN suspensions at pH values of 4.2 and different concentrations of particles.

The optical properties of the aqueous PPN suspensions were analyzed by UV-vis spectroscopy. The UV-vis absorption spectra were recorded at different PPN concentrations in the wavelength range of 300–1000 nm, as shown in Figure 4. PPN exhibits the typical π–π* transition band of the polypyrrole around 420 nm, in agreement with the literature [35,36]. At the same time, the observed band above ~650 nm is attributable to the sizeable π-conjugated structure of polypyrrole chains [37]. As expected, the absorbance increased proportionally to the PPN concentration. Similarly, Zha et al. reported that the absorbance increases linearly as the concentration of PPN in water is elevated, indicating the excellent dispersity of the aqueous PPN solution [15]. Furthermore, a calibration curve was obtained from this linear behavior, with a resulting extinction coefficient (*ԑ*) of 0.0559 a.u./(μg/mL∙cm) and a determination coefficient (R^2^) of 0.994 (Figure S3). It should be mentioned that the absorption spectra of PPN also depend on the nature of the solvent [38].

### 3.2. pH Effects over PPN

**Figure 5:**
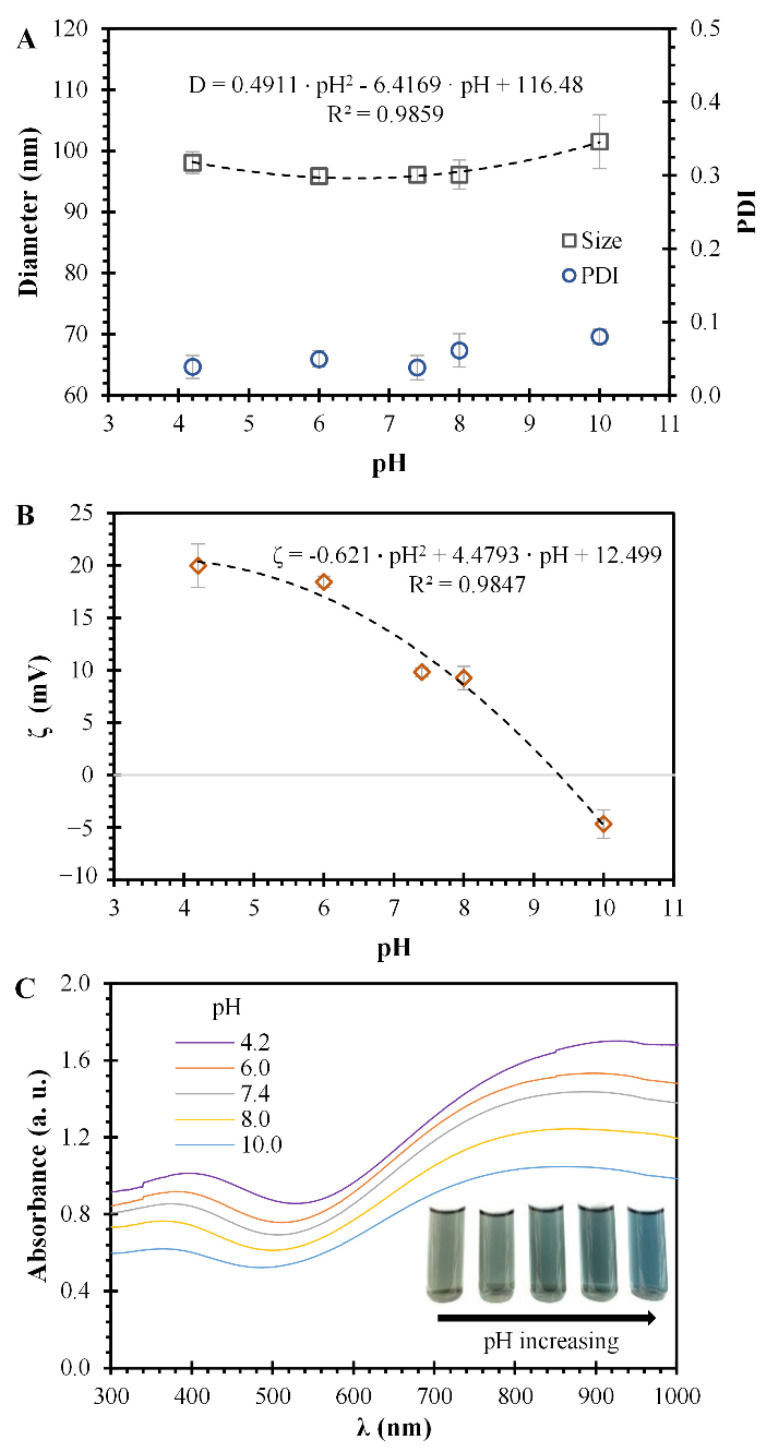
PPN properties at different pH values. (**A**) Diameter size distributions and PDI of PPN; Squares represent nanoparticle diameter sizes, the continuous line represents the quadrating fitting to the diameter size, and circles represent the polydispersity index. Means ± SD, *n* = 3. (**B**) ζ of PPN; squares represent nanoparticle ζ, and the continuous line represents the quadrating fitting. Means ± SD, *n* = 3. (**C**) Optical absorption spectra of PPN suspensions; insert shows the color of suspensions at different pH values, ranging from 4.2 to 10.

The pH effect over the hydrodynamic particle size, ζ, and optical spectra of PPN suspensions are presented in Figure 5. The pH values of PPN suspensions were: 4.2, 6.0, 7.4, 8.0, and 10.0. In this range of pH, the PPN size presents a quadratic behavior with respect to the pH of the suspension (Figure 5A). However, the size differences are not significant between the pH values except for a pH of 10, where a significant difference resulted, compared with all the other pH values. This behavior is similar to the PDI values obtained, the results of which indicate monodisperse particles (PDI < 0.1) at pH values below 10 (Figure 5A). As observed for the size results, the only significant difference in PDI was at a pH of 10, compared with all the other PDI values. The ζ results for the different pH suspensions could be described with a quadratic relationship, as presented in Figure 5B. The ζ values for suspensions with pH of 4.2 and 6.0 do not present significant variations, with values around 20 mV. However, when the pH value of the suspension increases to 7.4 and 8.0, the ζ decreases to values of 9.8 ± 0.4 and 9.3 ± 1.1 mV, correspondingly, with significant differences for the pH values of 4.2 and 6.0.

Nevertheless, the ζ differences between pH 7.4 and 8.0 are not significant. The ζ values of suspensions of PPN at pH 10 resulted in −4.7 ± 1.3 mV, with substantial differences from all the other ζ values. The changes in the ζ of PPN are related to the deprotonation process that suffers the polymeric chain of PPN as the pH rises [39,40]. This deprotonation affects the surface charge due to changes in the PPN surface composition. Other authors have reported that iron nanoparticles coated with polypyrrole change from a positive ζ value to a negative one at a pH higher than 9 [41,42]. The significant differences observed in size and PDI measurement results could be attributed to the change in the surface charge character of the PPN, from positive to negative, indicating that some disturbance was obtained by passing throughout the isoelectric point of PPN (~pH of 9.4).

The optical spectra of PPN at different pH values are presented in Figure 5C, with wavelength scans ranging from 300 to 1000 nm. The characteristic optical spectra bands of PPN suspensions are similar for all the pH values. However, when the pH of the PPN suspension increases, a shift to a shorter wavelength is produced in the spectra of PPN. Furthermore, a change in the color of the PPN solutions is observed, from a black-green color in acidic conditions to a light blue in alkaline conditions (insert of Figure 5C). These wavelength shifts are related to the surface charge of the PPN, as described in Figure 5B. As the PPN is deprotonated (pH rises), the hydroxyl groups cover the surface, and a shift to shorter wavelengths is observed.

### 3.3. Determination of Model Parameters: η,U, and $S_{W}$

The order of magnitude values of η (adiabatic cells) and U were 0.65 and 7 W/m^2^·K, respectively. These values were used to initiate the minimization of the sum of squares of differences between the experimental and predicted temperature increments. The resulting value of η was considered constant for all the nanoparticle concentrations. This consideration is justified by the low PPN polydispersity (monodisperse particles), the homogeneous nanoparticle morphology, and the range of particle concentrations used [21,43]. The parameter U changed proportionally to the nanoparticle concentration. This effect could be explained by the coefficient U’s dependence on the media’s temperature after irradiation [27].

The resulting value η, from the minimization of the sum of squares, was 0.68. This value is greater than other similar systems of PPN prepared using PVP and the same oxidant [2,5] and similar to gold nanoparticles of similar sizes [44]. Other works using FeCl_3_ as oxidant have shown remaining Fe at the PPN surface in the form of iron oxides, improving the electrical conductivity and optical properties of the particles by the formation of a semiconductor–semiconductor heterojunction [45]. For instance, these remaining oxides could also improve the η value. The values of U for different PPN concentrations in the suspension are shown in Table 2. Furthermore, a value of 4.7 × 10^5^ (W/m^3^) was fitted to $S_{W}$ by simulation of the particle-free suspension. These values agree with other values reported in the literature [46,47].

#### 3.3.1. Photothermal Effect of PPN at Different Concentrations

**Figure 6:**
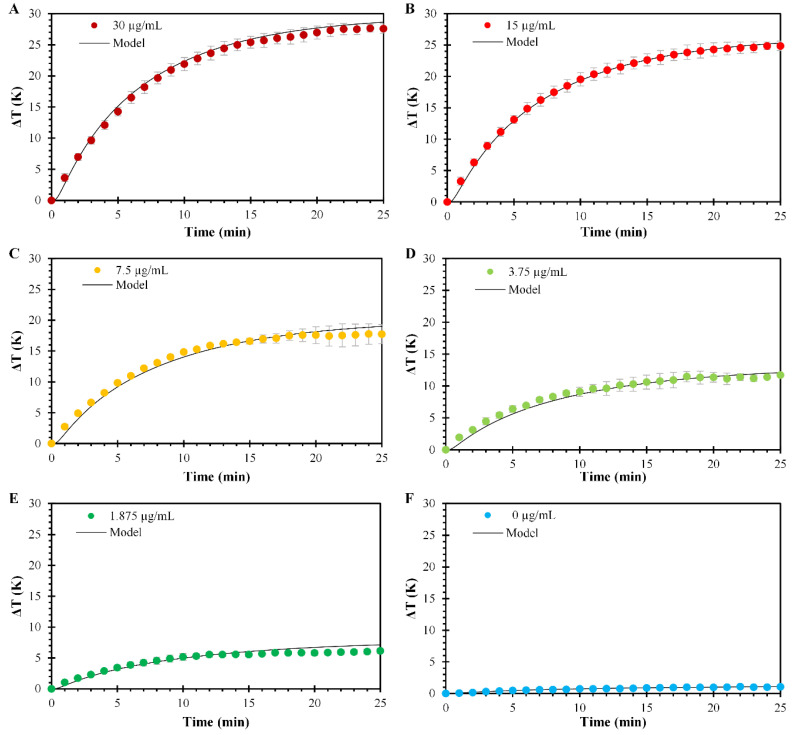
Temperature profiles of PPN suspensions at pH values of 7.4 and model fitting at concentrations of (**A**) 30, (**B**) 15, (**C**) 7.5, (**D**) 3.75, (**E**) 1.875, and (**F**) 0 µg/mL. Circles represent experimental data, and the continuous solid lines represent the model fitting. Means ± SD, *n* = 3.

The photothermal effect of PPN at different concentrations under laser irradiation and the respective heat transfer model fittings are presented in Figure 6. The heating analysis as a function of PPN concentration was evaluated at a fixed pH value of 7.4 to simulate biological conditions. The concentrations of PPN evaluated were 30, 15, 7.5, 3.75, 1.875, 0 µg/mL, equivalent to the absorbances of 1.677, 0.839, 0.419, 0.210, and 0.105, respectively (Figure 6A–E). The spatiotemporal temperature distributions of irradiated PNP suspensions show the same heating profile, but different temperature increments are reached proportionally to the nanoparticle concentration increase. The profiles started with a period of rapid heating until a plateau was reached, indicating a steady state. Results show a direct dependency between the PPN concentration and the temperature increment obtained, with maximum temperature increments of 27.6, 24.8, 17.7, 11.7, and 6 °C for 30, 15, and 7.5, 3.75, and 1.875 µg/mL, respectively. Furthermore, a slight temperature increment for the 0 µg/mL solution is presented after irradiation, confirming that most of the temperature increments are due to the laser interactions with the PPN. Comparable results have been reported in the literature for the spatiotemporal temperature distributions of other irradiated materials, such as gold nanoparticles [48], gold nanorods [24], carbon nanotubes [49], iron oxide nanoparticles [50], etc.

**Figure 7:**
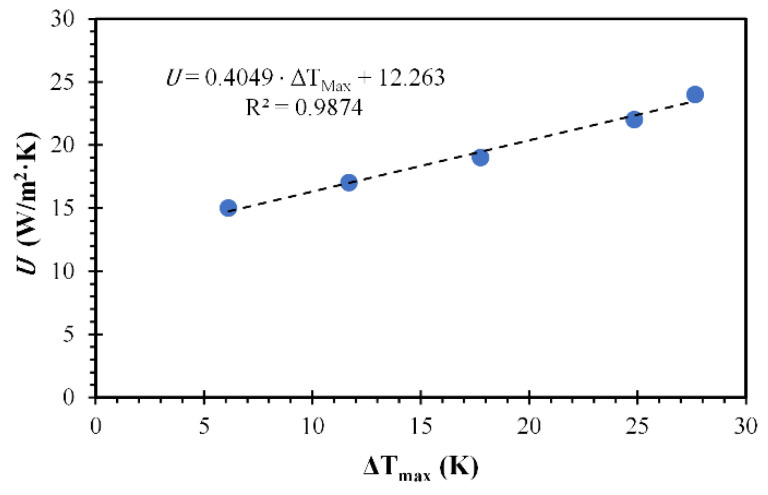
Estimated overall heat transfer coefficient. Circles represent the overall heat transfer coefficient, and the continuous dotted line represents a linear fitting.

The mathematical analysis for the spatiotemporal temperature distributions of irradiated PNP at different concentrations shows good agreement with the experimental data. The model predicted the temperatures with an average absolute error below 1 °C, compared to the experimental data, as shown in Figure S4. The best agreement is found at higher PPN suspension concentrations. However, with lower nanoparticle concentrations, the model underestimates at the early stages of heating, overestimating the temperature change when reaching the thermal plateau. Furthermore, a linear correlation between the overall heat transfer coefficient and temperature increment is shown in Figure 7, contrasting with the non-linear dependency predicted by the correlation used as a starting point (Figure S2).

#### 3.3.2. Photothermal Effect of PPN at Different pH

**Figure 8:**
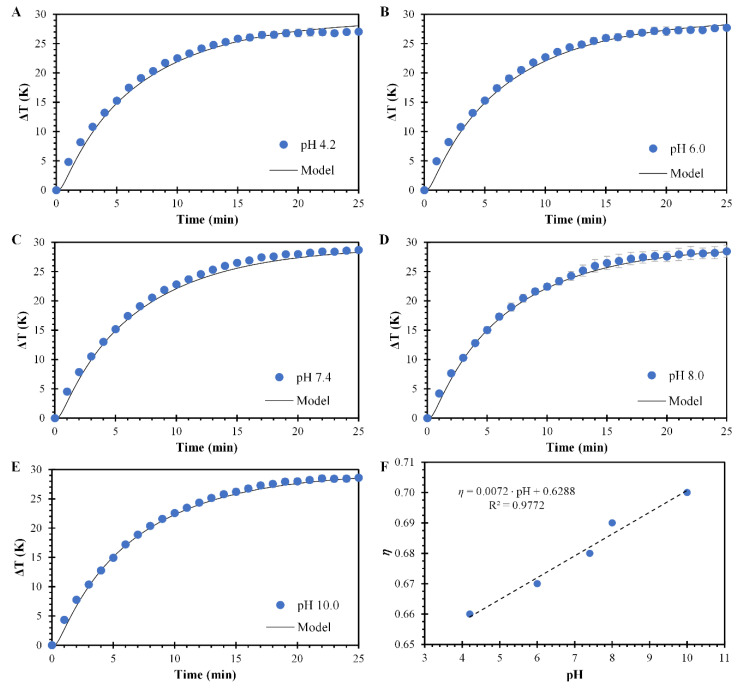
Laser irradiation effects of PPN suspensions as a function of the pH. Temperature profiles of PPN suspensions and model fitting at pH values of (**A**) 7.4, (**B**) 6.0, (**C**) 7.4, (**D**) 8.0, and (**E**) 10. Circles represent the experimental data of PPN suspensions, and the continuous solid lines represent model fitting. Means ± SD, *n* = 3. (**F**) The efficiency of transducing resonant irradiation of light to heat (η) for a PPN suspension of 28 µg/mL as a function of the pH. Circles represent the η value obtained, and the dotted line the linear adjustment.

The photothermal effect of PPN at different pH was performed at an average nanoparticle concentration of 28.5 µg/mL. Figure 8A–E present the measured temperature profiles and their model predictions at solution pH values of 4.2, 6.0, 7.4, 8.0, and 10, respectively. The mathematical analysis at different pH values agrees well with the experimental data. The model predicted the temperatures with an average absolute error below 1 °C compared to the experimental data (Figure S5). The heating profiles in Figure 8 present similar behavior within them, observing a gradual temperature increase until a steady temperature is reached.

Furthermore, a small increment in the suspensions’ maximum temperature occurs when pH increases. This increment in the maximum temperature is not related to the absorbance of the suspensions but to variations in the efficiency of transducing resonant irradiation of light to heat. As a result, the model predicts that this efficiency increases linearly with the pH values of the suspension (Figure 8F). This finding is supported by the ζ value measurements presented in Figure 5B, in which a decrease in the ζ value occurs as the pH increases, indicating that the nanoparticle surface charge affects the transduction of light.

**Figure 9:**
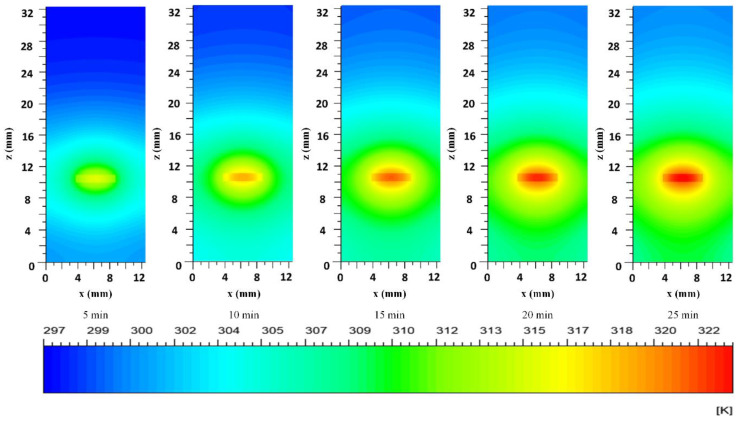
Computed temperature contours at different times during the suspensions heating at PPN of 3.75 µg/mL and pH of 7.4. Plane xz at y = 6.3 mm located at the midpoint of the cuvette depth. The color scale represents temperatures in K.

The predicted temperature contours for a concentration of PPN of 3.75 µg/mL and pH 7.4 are presented in Figure 9. At a glance, no matter which contour is selected, the laser region is the hottest spot in the contour, unlike the suspension/air interface in the suspension region, which remains almost at room temperature during the experiment. The temperature of ~51 °C is reached in the laser region, with an exposure time of 25 min. The contour analysis helps to understand and control the maximum temperature reached in the irradiated volume as a function of time, which is necessary for all biological applications to avoid undesirable effects, for example, necrosis, damage surrounding healthy tissue, and the possible effects of prolonged laser exposure.

## 4. Conclusions

Polypyrrole nanoparticles (PPN) were synthesized by oxidative chemical polymerization, resulting in an appropriate size, good stability, and strong absorption in the NIR region, suitable for applications in photothermal therapies. The zeta potential (ζ) of PPN could be tuned from positive to negative values by controlling the pH of the suspensions while maintaining similar sizes. A three-dimensional mathematical model that considers the extinct radiation of suspensions of PPN and the photothermal transduction efficiency was developed. A direct approach was proposed to determine the order of magnitude of the photothermal transduction efficiency and the overall heat transfer coefficient. The heat transfer model shows good agreement between the experimental and the predicted temperature changes. Furthermore, a linear dependency of the overall heat transfer coefficient with the temperature was found. For PPN of 60 nm dispersed at a pH of 7.4, the photothermal transduction efficiency had a value of 0.68.

Additionally, a linear dependency was found between the photothermal transduction efficiency and the pH of the suspensions. The model could predict temperature zones with potential photothermal therapy use around the laser region regarding nanoparticle concentration and power. The current approach for modeling the conversion of NIR light into heat by using nanoparticle suspensions could contribute to the analysis and design of systems/devices for photothermal ablation of cells and tissues.

## Figures and Tables

**Table 1:** Main physical parameters used in the mathematical model.

Parameter	Description	Unit	Value
ρ	Dispersion density	$\mathrm{kg}/m^{3}$	998.2
$C_{P}$	Heat capacity at constant pressure	J/kg·K	4182
k	Thermal conductivity	W/m·K	0.6
$I_{\mathrm{laser}}$	Laser power	W	1.5
$V_{\mathrm{laser}}$	Irradiated volume	$m^{3}$	6.25 × 10^−8^
$T_{f}$	Temperature of surrounding media (air)	K	297.15
$T_{\infty }$	Temperature of the domain exterior	K	297.15
$\varepsilon_{\mathrm{cell}}$	Emissivity of the cell walls	−	0.925 *
$\varepsilon_{\mathrm{water}}$	Emissivity of the air/solution interface	−	0.96 *

* The emissivity of glass at 300 K is between 0.90 and 0.95; the emissivity of water at 300 K is 0.96 [27].

**Table 2:** Maximum temperature increments and the corresponding values of U as a function of the PPN concentration.

PPN Concentration (µg/mL)	$\Delta T_{max}$ (K)	U $\left(W/m^{2}\cdot K\right)$
30	27.7	24
15	24.9	22
7.5	17.8	19
3.75	11.7	17
1.875	6.1	15

## Data Availability

Not applicable.

## Supplementary Materials

The following supporting information can be downloaded at: https://www.mdpi.com/article/10.3390/polym14153151/s1, Figure S1: Temperature gradients at the thermocouple position (6.25, 6.25, 16 mm) for the adiabatic cell for different photothermal transduction efficiency (η). The experimental data correspond to a concentration of PPN of 30 µg/mL and a pH value of 7.4; Figure S2: Calculated temperature gradients for different values of overall heat transfer coefficient (U). The solid dots correspond to the experimental heating data at a concentration of PPN of 30 µg/mL and a pH value of 7.4; Figure S3: PPN calibration curve at a wavelength of 808 nm. The mass extinction coefficient (ε) of PPN is 0.0559 $a.u./\left(\frac{\mu g}{mL}\cdot cm\right)$. Mean ±SD, *n* = 3; Figure S4: Comparison of simulated temperature gradients and experimental temperature gradients for the different concentrations of PPN at a pH value of 7.4; Figure S5: Comparison of simulated temperature gradients and experimental temperature gradients for the different pH values at a concentration of PPN of 30 µg/mL.

## References

[1] Zhou J., Lu Z., Zhu X., Wang X., Liao Y., Ma Z., Li F. (2013). NIR Photothermal Therapy Using Polyaniline Nanoparticles. Biomaterials.

[2] Chen M., Fang X., Tang S., Zheng N. (2012). Polypyrrole Nanoparticles for High-Performance in vivo near-Infrared Photothermal Cancer Therapy. Chem. Commun..

[3] Baez-Castillo L., Ortiz-Rascón E., Carrillo-Torres R.C., Bruce N.C., Garduño-Mejia J., Lucero-Acuña A., Álvarez-Ramos M.E. (2021). Deep Photothermal Effect Induced by Stereotactic Laser Beams in Highly Scattering Media. Opt. Lett..

[4] Yang J., Zhai S., Qin H., Yan H., Xing D., Hu X. (2018). NIR-Controlled Morphology Transformation and Pulsatile Drug Delivery Based on Multifunctional Phototheranostic Nanoparticles for Photoacoustic Imaging-Guided Photothermal-Chemotherapy. Biomaterials.

[5] Guo B., Zhao J., Wu C., Zheng Y., Ye C., Huang M., Wang S. (2019). One-Pot Synthesis of Polypyrrole Nanoparticles with Tunable Photothermal Conversion and Drug Loading Capacity. Colloids Surfaces B Biointerfaces.

[6] Balint R., Cassidy N.J., Cartmell S.H. (2014). Conductive Polymers: Towards a Smart Biomaterial for Tissue Engineering. Acta Biomater..

[7] Liu Y., Yu Q., Chang J., Wu C. (2019). Nanobiomaterials: From 0D to 3D for Tumor Therapy and Tissue Regeneration. Nanoscale.

[8] Vines J.B., Lim D.J., Park H. (2018). Contemporary Polymer-Based Nanoparticle Systems for Photothermal Therapy. Polymers.

[9] Vaitkuviene A., Kaseta V., Voronovic J., Ramanauskaite G., Biziuleviciene G., Ramanaviciene A., Ramanavicius A. (2013). Evaluation of Cytotoxicity of Polypyrrole Nanoparticles Synthesized by Oxidative Polymerization. J. Hazard. Mater..

[10] Xiao Z., Xu C., Jiang X., Zhang W., Peng Y., Zou R., Huang X., Liu Q., Qin Z., Hu J. (2016). Hydrophilic Bismuth Sulfur Nanoflower Superstructures with an Improved Photothermal Efficiency for Ablation of Cancer Cells. Nano Res..

[11] Wang J., Lin F., Chen J., Wang M., Ge X. (2015). The Preparation, Drug Loading and in vitro NIR Photothermal-Controlled Release Behavior of Raspberry-like Hollow Polypyrrole Microspheres. J. Mater. Chem. B.

[12] Song X., Chen Q., Liu Z. (2015). Recent Advances in the Development of Organic Photothermal Nano-Agents. Nano Res..

[13] Yang K., Xu H., Cheng L., Sun C., Wang J., Liu Z. (2012). In vitro and in vivo Near-Infrared Photothermal Therapy of Cancer Using Polypyrrole Organic Nanoparticles. Adv. Mater..

[14] Chen J., Ning C., Zhou Z., Yu P., Zhu Y., Tan G., Mao C. (2019). Nanomaterials as Photothermal Therapeutic Agents. Prog. Mater. Sci..

[15] Zha Z., Yue X., Ren Q., Dai Z. (2013). Uniform Polypyrrole Nanoparticles with High Photothermal Conversion Efficiency for Photothermal Ablation of Cancer Cells. Adv. Mater..

[16] Hong J.Y., Yoon H., Jang J. (2010). Kinetic Study of the Formation of Polypyrrole Nanoparticles in Water-Soluble Polymer/Metal Cation Systems: A Light-Scattering Analysis. Small.

[17] Andreozzi A., Brunese L., Iasiello M., Tucci C., Vanoli G.P. (2019). Modeling Heat Transfer in Tumors: A Review of Thermal Therapies. Ann. Biomed. Eng..

[18] Singh S., Melnik R. (2020). Thermal Ablation of Biological Tissues in Disease Treatment: A Review of Computational Models and Future Directions. Electromagn. Biol. Med..

[19] Qin Z., Bischof J.C. (2012). Thermophysical and Biological Responses of Gold Nanoparticle Laser Heating. Chem. Soc. Rev..

[20] Roper D.K., Ahn W., Hoepfner M. (2007). Microscale Heat Transfer Transduced by Surface Plasmon Resonant Gold Nanoparticles. J. Phys. Chem. C.

[21] Tian Q., Jiang F., Zou R., Liu Q., Chen Z., Zhu M., Yang S., Wang J., Wang J., Hu J. (2011). Hydrophilic Cu_9_S_5_ Nanocrystals: A Photothermal Agent with a 25.7% Heat Conversion Efficiency for Photothermal Ablation of Cancer Cells in Vivo. ACS Nano.

[22] Baffou G., Cichos F., Quidant R. (2020). Applications and Challenges of Thermoplasmonics. Nat. Mater..

[23] Liu Y., Kangas J., Wang Y., Khosla K., Pasek-Allen J., Saunders A., Oldenburg S., Bischof J. (2020). Photothermal Conversion of Gold Nanoparticles for Uniform Pulsed Laser Warming of Vitrified Biomaterials. Nanoscale.

[24] Huang H.C., Rege K., Heys J.J. (2010). Spatiotemporal Temperature Distribution and Cancer Cell Death in Response to Extracellular Hyperthermia Induced by Gold Nanorods. ACS Nano.

[25] Zayan S.E., El-Shazly A.H., El-Kady M.F., Haider A.J., Jabur A.R., Salame C., Vokas G. Assessment of Polypyrrole Nanoparticles Synthesized in Presence and Absence of Surfactant for Heavy Metals Decontamination. Proceedings of the AIP Conference Proceedings, Technologies and Materials for renewable Energy, Environment and Sustainability: TMREES19Gr, Athens, Greece, 4–6 September 2019.

[26] Wang M. (2016). Emerging Multifunctional NIR Photothermal Therapy Systems Based on Polypyrrole Nanoparticles. Polymers.

[27] Incropera F.P., DeWitt D.P. (1999). Fundamentos de Transferencia de Calor.

[28] Wen J., Tian Y., Mei Z., Wu W., Tian Y. (2017). Synthesis of Polypyrrole Nanoparticles and Their Applications in Electrically Conductive Adhesives for Improving Conductivity. RSC Adv..

[29] Pan H., Marsh J.N., Christenson E.T., Soman N.R., Ivashyna O., Lanza G.M., Schlesinger P.H., Wickline S.A., Abelson J.N., Simon M.I. (2012). Postformulation Peptide Drug Loading of Nanostructures. Methods in Enzymology.

[30] Samanta D., Meiser J.L., Zare R.N. (2015). Polypyrrole Nanoparticles for Tunable, pH-Sensitive and Sustained Drug Release. Nanoscale.

[31] Liu H., Li W., Cao Y., Guo Y., Kang Y. (2018). Theranostic Nanoplatform Based on Polypyrrole Nanoparticles for Photoacoustic Imaging and Photothermal Therapy. J. Nanopart. Res..

[32] Kisiel A., Korol D., Michalska A., Maksymiuk K. (2021). Polypyrrole Nanoparticles of High Electroactivity. Simple Synthesis Methods and Studies on Electrochemical Properties. Electrochim. Acta.

[33] Zhang H., Zhong X., Xu J., Chen H. (2008). Fe_3_O_4_/Polypyrrole/Au Nanocomposites with Core/Shell/Shell Structure: Synthesis, Characterization, and Their Electrochemical Properties. Langmuir.

[34] Tiwari A.P., Hwang T.I., Oh J.M., Maharjan B., Chun S., Kim B.S., Joshi M.K., Park C.H., Kim C.S. (2018). PH/NIR-Responsive Polypyrrole-Functionalized Fibrous Localized Drug-Delivery Platform for Synergistic Cancer Therapy. ACS Appl. Mater. Interfaces.

[35] Liu Y., Chu Y., Yang L. (2006). Adjusting the Inner-Structure of Polypyrrole Nanoparticles through Microemulsion Polymerization. Mater. Chem. Phys..

[36] Zeng G., An Y., Fei H., Yuan T., Qing S., Ci L., Xiong S., Feng J. (2018). Green and Facile Synthesis of Nanosized Polythiophene as an Organic Anode for High-Performance Potassium-Ion Battery. Funct. Mater. Lett..

[37] Li X.-G., Li A., Huang M.-R., Liao Y., Lu Y.-G. (2010). Efficient and Scalable Synthesis of Pure Polypyrrole Nanoparticles Applicable for Advanced Nanocomposites and Carbon Nanoparticles. J. Phys. Chem. C.

[38] Hazarika J., Kumar A. (2013). Controllable Synthesis and Characterization of Polypyrrole Nanoparticles in Sodium Dodecylsulphate (SDS) Micellar Solutions. Synth. Met..

[39] Zhang X., Bai R. (2003). Surface Electric Properties of Polypyrrole in Aqueous Solutions. Langmuir.

[40] Pei Q., Qian R. (1991). Protonation and Deprotonation of Polypyrrole Chain in Aqueous Solutions. Synth. Met..

[41] Shanehsaz M., Seidi S., Ghorbani Y., Shoja S.M.R., Rouhani S. (2015). Polypyrrole-Coated Magnetic Nanoparticles as an Efficient Adsorbent for RB19 Synthetic Textile Dye: Removal and Kinetic Study. Spectrochim. Acta-Part A Mol. Biomol. Spectrosc..

[42] Bai L., Li Z., Zhang Y., Wang T., Lu R., Zhou W., Gao H., Zhang S. (2015). Synthesis of Water-Dispersible Graphene-Modified Magnetic Polypyrrole Nanocomposite and Its Ability to Efficiently Adsorb Methylene Blue from Aqueous Solution. Chem. Eng. J..

[43] Alrahili M., Peroor R., Savchuk V., McNear K., Pinchuk A. (2020). Morphology Dependence in Photothermal Heating of Gold Nanomaterials with Near-Infrared Laser. J. Phys. Chem. C.

[44] Jiang K., Smith D.A., Pinchuk A. (2013). Size-Dependent Photothermal Conversion Efficiencies of Plasmonically Heated Gold Nanoparticles. J. Phys. Chem. C.

[45] Gogoi R., Singh A., Moutam V., Sharma L., Sharma K., Halder A., Siril P.F. (2022). Revealing the Unexplored Effect of Residual Iron Oxide on the Photoreforming Activities of Polypyrrole Nanostructures on Plastic Waste and Photocatalytic Pollutant Degradation. J. Environ. Chem. Eng..

[46] Mirrahimi M., Hosseini V., Kamrava S.K., Attaran N., Beik J., Kooranifar S., Ghaznavi H., Shakeri-Zadeh A. (2018). Selective Heat Generation in Cancer Cells Using a Combination of 808 nm Laser Irradiation and the Folate-Conjugated Fe_2_O_3_ @Au Nanocomplex. Artif. Cells Nanomed. Biotechnol..

[47] Sun C., Ji C., Li Y., Kuang J., Wu J. (2021). A Comparison Study of Photothermal Effect between Moxibustion Therapy and Laser Irradiation on Biological Tissue. Int. J. Therm. Sci..

[48] Lu J., Cai L., Dai Y., Liu Y., Zuo F., Ni C., Shi M., Li J. (2021). Polydopamine-Based Nanoparticles for Photothermal Therapy/Chemotherapy and Their Synergistic Therapy with Autophagy Inhibitor to Promote Antitumor Treatment. Chem. Rec..

[49] Zhao Y., Zhao T., Cao Y., Sun J., Zhou Q., Chen H., Guo S., Wang Y., Zhen Y., Liang X.J. (2021). Temperature-Sensitive Lipid-Coated Carbon Nanotubes for Synergistic Photothermal Therapy and Gene Therapy. ACS Nano.

[50] Estelrich J., Antònia Busquets M. (2018). Iron Oxide Nanoparticles in Photothermal Therapy. Molecules.

