# Supplementary material for: Spatiotemporal Temperature Distribution of NIR Irradiated Polypyrrole Nanoparticles and Effects of pH
Source: Polymers (Basel). 2022 Aug 2;14(15):3151. doi: 10.3390/polym14153151 (PMC9371108; doi:10.3390/polym14153151)
Supplement: Supplementary file 1 [file polymers-14-03151-s001.zip › polymers-1847368-supplementary.pdf]

Supplementary:

Spatiotemporal Temperature Distribution of NIR Irradiated Polypyrrole Nanoparticles and Effects of pH

Omar Peñuñuri-Miranda [1], Miguel Olivas-Martinez [1], José Alberto Ibarra-Espinoza [1], Rosalva Josefina Rodríguez-Córdova [1,2], Karol Yesenia Hernández-Giottonini [1,2], Daniel Fernández-Quiroz [1], Paul Zavala-Rivera [1,2] and Armando Lucero-Acuña [1,2]*

[1] Department of Chemical and Metallurgical Engineering, University of Sonora, Hermosillo, SON 83000, Mexico; a212205033@unison.mx (O.P.-M.); miguel.olivas@unison.mx (M.O.-M.); a217201103@unison.mx (J.A.I.-E.); rosalva.rodriguez@unison.mx (R.J.R.-C.); yesenia.hgio@hotmail.com (K.Y.H.-G.); daniel.fernandez@unison.mx (D.F.-Q.); paul.zavala@unison.mx (P.Z.-R.);

[2] Nanotechnology Graduate Program, Department of Physics, University of Sonora, Hermosillo, SON 83000, Mexico

* Correspondence: armando.lucero@unison.mx (A.L.-A.); Tel.: +52 662 259 2105

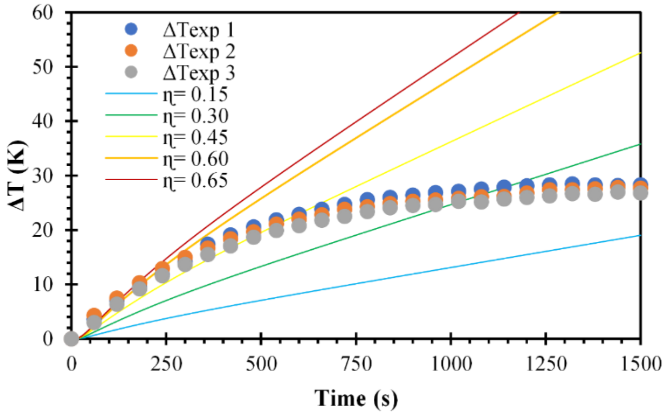

Figure S1. Temperature gradients at the thermocouple position (6.25 mm, 6.25 mm, 16 mm) for the adiabatic cell for different photothermal transduction efficiency (η). The experimental data corresponds to a concentration of PPN of 30 μg/mL and a pH value of 7.4.

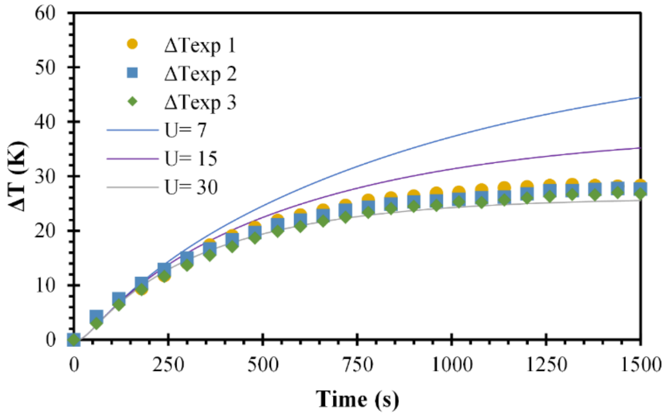

Figure S2. Calculated temperature gradients for different values of overall heat transfer coefficient (U). The solid dots correspond to the experimental heating data at a concentration of PPN of 30 μg/mL and a pH value of 7.4.

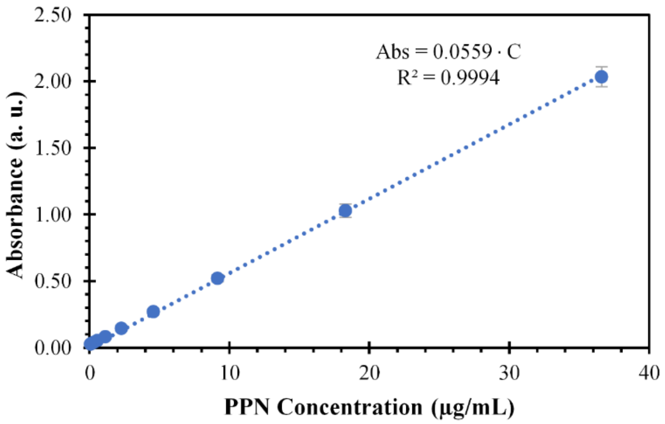

Figure S3. PPN calibration curve at a wavelength of 808 nm. The mass extinction coefficient (ε) of PPN is 0.0559 $a.u./\left(\frac{\mu g}{mL} \cdot cm\right)$. Mean ±SD, n=3.

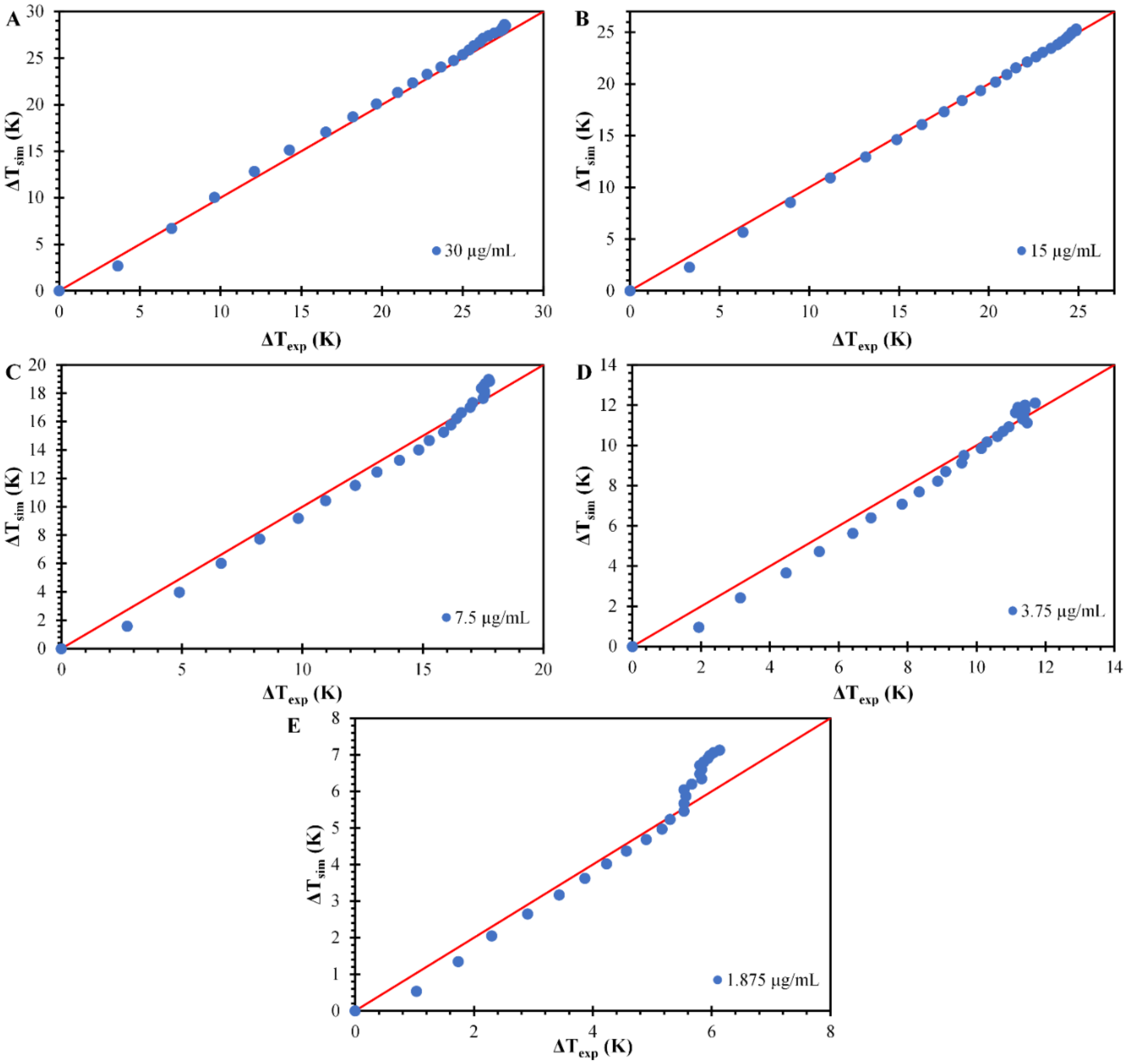

Figure S4. Comparative of simulated temperature gradients and experimental temperature gradients for the different concentrations of PPN at a pH value of 7.4.

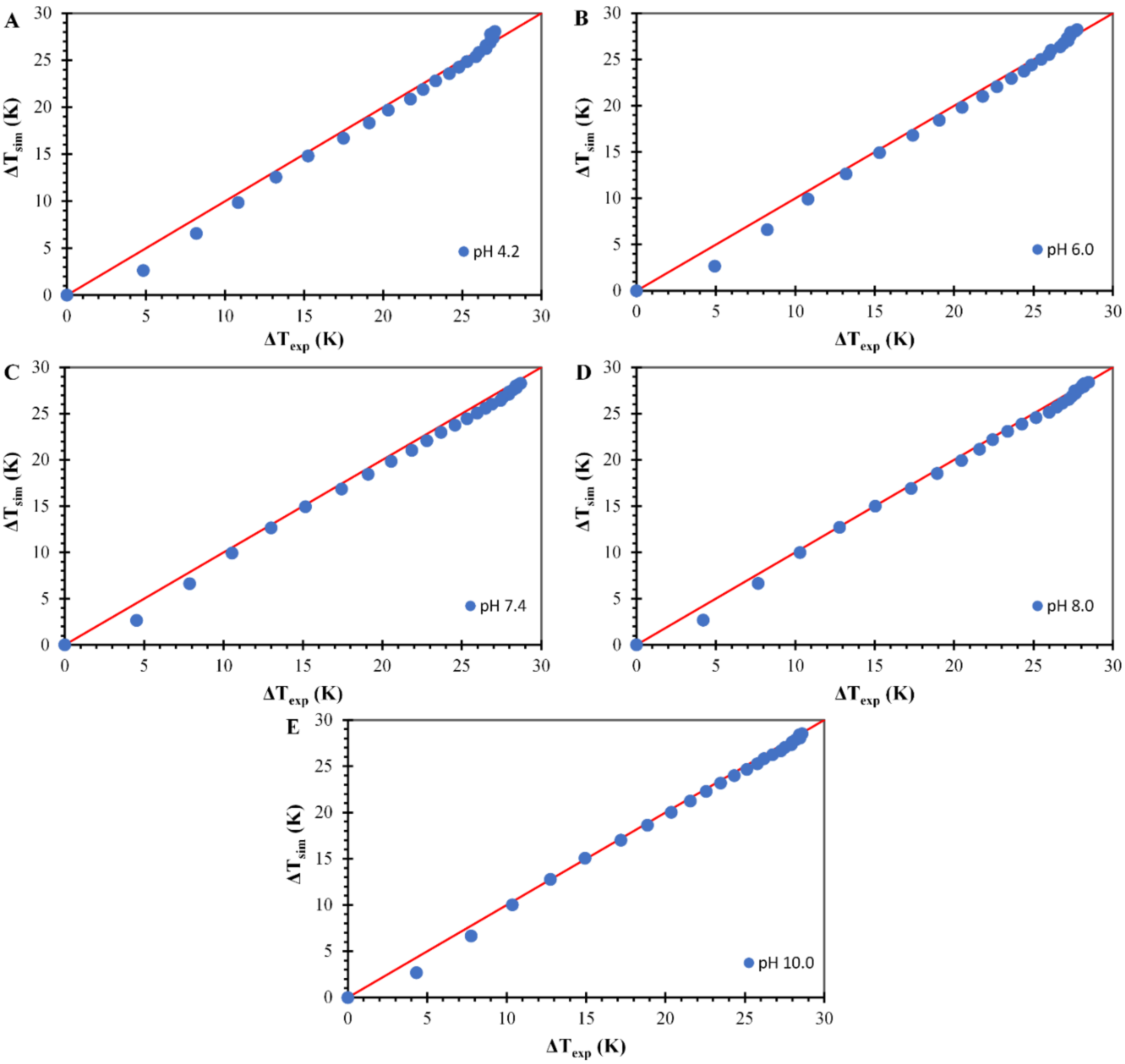

Figure S5. Comparative of simulated temperature gradients and experimental temperature gradients for the different pH values at a concentration of PPN of 30 μg/mL.
